# A role for the terminal C5-C9 complement pathway in idiopathic pulmonary fibrosis

**DOI:** 10.3389/fmed.2023.1236495

**Published:** 2023-08-09

**Authors:** Liv I. B. Sikkeland, Thor Ueland, May B. Lund, Michael Thomas Durheim, Tom Eirik Mollnes

**Affiliations:** ^1^Faculty of Medicine, Institute of Clinical Medicine, University of Oslo, Oslo, Norway; ^2^Department of Respiratory Medicine, Oslo University Hospital Rikshospitalet, Oslo, Norway; ^3^Institute of Internal Medicine, Oslo University Hospital, University of Oslo, Oslo, Norway; ^4^K. G. Jebsen, Thrombosis Research Center, University of Tromsø, Tromsø, Norway; ^5^Department of Immunology, Oslo University Hospital Rikshospitalet, University of Oslo, Oslo, Norway; ^6^Research Laboratory, Nordland Hospital, Bodø, Norway; ^7^Centre of Molecular Inflammation Research, Norwegian University of Science and Technology, Trondheim, Norway

**Keywords:** interstitial lung disease, idiopathic pulmonary fibrosis, terminal complement complex, proteomics, bronchial lavage fluid

## Abstract

Idiopathic pulmonary fibrosis (IPF) is a chronic progressive interstitial lung disease characterized by damage to the alveolar epithelium, leading to fibrosis and excessive accumulation of extracellular matrix in the interstitium of the lung. In the present study we performed high-resolution proteomic profiling of bronchoalveolar lavage (BAL) from IPF patients and controls, and found that the complement pathway was highly upregulated in IPF. The proteins C5, C6, C7, C8, and C9, all of which are part of the complement end product, TCC, were all upregulated. We also found that TCC levels were increased in plasma among IPF patients compared to controls, after adjustment for age, sex and BMI [mean (SD) 0.62 (0.24) vs. 0.33 (0.10), *p* = 0.031]. These findings suggest a role for the complement system in the pathogenesis of IPF.

## Introduction

Idiopathic pulmonary fibrosis (IPF) is a chronic progressive interstitial lung disease that leads to decline in lung function and early mortality (1). Although anti-fibrotic therapies approved for IPF improve respiratory outcomes (2, 3), these drugs are associated with substantial side effects, and do not cure the disease. The prognosis for patients with IPF remains poor, with median survival often estimated to approximately 3–5 years (1).

The pathogenesis of IPF is multifactorial and characterized by progressive fibrosis and excessive accumulation of extracellular matrix in the interstitium of the lung, with an imbalance between anti-fibrotic and pro-fibrotic factors leading to collagen deposition (4).

To identify proteins involved in these processes, we performed high-resolution proteomic profiling of bronchoalveolar lavage (BAL) from IPF patients and controls, and identified the complement system to be differentially regulated. The objectives were to (i) categorize the complement factors identified by proteomics, and (ii) assess the clinical significance of circulating soluble terminal C5b-9 complement complex (TCC) in plasma, a key downstream readout of systemic complement activation, and its association with lung function and complement levels in BAL.

## Materials and methods

### Subjects

Twenty-nine patients with IPF [mean age (SD) 66 (7) years, 6 females], and 10 controls [aged 48 (13) years, 7 females] were included in this cross-sectional study and underwent bronchoscopy with BAL (5). IPF patients were diagnosed according to ATS/ERS criteria after multidisciplinary evaluation (6). Controls underwent bronchoscopy as part of planned surveillance >6 months after resection of carcinoid tumor, and were considered healthy without signs of lung disease. Contraindications for BAL for all subjects were forced vital capacity (FVC) <50% predicted and/or diffusing capacity of the lung for carbon monoxide (DLCO) < 40% predicted using GLI reference values (7, 8). These contraindications will exclude patients with severe IPF since the BAL procedure entails too high risk of complications in this group (9). We excluded patients on anti-fibrotic treatment, active smokers during the last year, and those aged >75 years. Characteristics of the study population are presented in Table 1.

**Table 1:** Characteristics of the study cohort.

	IPF (*n* = 29)	Controls (*n* = 10)
Age (years) Age (years)	66 (7)	48 (14)
Sex (female/male)	6/23	7/3
BMI (kg/m^2^)	28.7 (3.8)	25.6 (4.45)
Lung function		
FVC (L)	3.2 (0.6)	4.3 (0.7)
FVC % predicted	74 (14)	104 (15)
FEV1 (L)	2.6 (0.5)	3.4 (0.5)
FEV1% predicted	78 (14)	105 (16)
DLCO (mmol/min*kPa)	4.6 (0.8)	8.2 (2)
DLCO % predicted	52 (8)	99 (13)
Blood samples		
Hb (g/dL)	14.4 (1.3)	13.9 (1.3)
CRP (mg/L)	4 (4.2)	1.4 (1.4)
Leukocytes (10^6^/L)	8 (2)	5 (1)
Cells in BAL		
Macrophages (%)	80 (15)	83 (17)
Neutrophils (%)	6 (8)	7 (18)
Lymphocytes (%)	9 (9)	10 (11)
Eosinophils (%)	4 (5)	0 (0)

IPF, idiopathic pulmonary fibrosis; BMI, body mass index; FVC, Forced vital capacity; FEV1, forced expiratory volume-one second; DLCO, diffusing capacity for carbon monoxide; Hb, hemoglobin; CRP, C-reactive protein. Data are presented as mean (SD).

### Bronchoalveolar lavage

Bronchoalveolar lavage (BAL) collection and preparation were performed as described previously (5). In short, fiberoptic bronchoscopy was performed in local anesthesia with 10% lidocaine with the subject in a supine position. Alfentanil (0.25–1.0 mg) was given intravenously in some subjects. With the bronchoscope wedged in a middle lobe segment, BAL was performed by instillation of 3 × 40 mL aliquots of Ringer solution (Fresenius Kabi, Germany) at 37°C. After each instillation, aspiration was performed with a negative pressure of 10–12 mmHg, until backflow stopped or the patient started to cough. The recoveries from the second and third aliquot were used in this study. BAL was filtered through a nylon web (pore size; 48 μm) and centrifuged at 380 g for 5 min at 4°C. The cell pellet was re-suspended in 1 mL phosphate-buffered saline and supernatant was snap frozen in liquid nitrogen and stored at −80°C. Total cell count and viability were determined with tryptan blue staining and a hematological cell counter. Twenty-two of the 29 IPF patients had BAL samples with recovery of >30% of instillation, >95% viable cells and < 5% epithelial cells (10).

### Proteomics in BAL

Sample preparation for mass spectroscopy was performed as described (11) using BAL supernatant from 22 IPF patients and 10 controls. The peptide samples were analyzed by liquid chromatography–tandem mass spectrometry (nEasy-LC coupled to QExactive Plus, Thermo) with 60 min separation gradient. One replicate was performed per sample. MaxQuant v1.6.1.0 was used for protein identification and label-free quantification. Perseus v1.6.1.3 was used for statistical analysis based on normalized intensities (*t*-test with FDR < 0.05). The mass spectrometry proteomics data were deposited to the ProteomeXchange Consortium PRIDE repository with identifier PXD036638 (12). In the supplement, the mass spectrometry data is available as a raw data file (excel file) with analysis from Perseus. Pathway analyses were performed using Cytoscape 3.8.0 with cytoKEGG. Proteomic data were adjusted for age and sex.

### EDTA plasma and TCC analysis

Blood (EDTA) was collected <2 h before BAL collection. EDTA plasma was centrifuged (1,400 × *g*, 20 min) and plasma was snap frozen in liquid nitrogen and stored at −80°C. The fluid-phase terminal complement complex (TCC), consisting of the components C5b, C6, C7, C8, and C9, was measured by ELISA using a monoclonal antibody aE11 as originally described in (13), and later modified by using another detection antibody (14). The upper reference value for TCC in plasma was 0.7 complement arbitrary units (CAU)/mL (14). This value was determined using 40 healthy blood donors (20 females and 20 males), using the 95-percentile for defining the cut-off. A detailed protocol for the TCC ELISA is presented in Supplementary material.

### Statistical analysis

Comparison of complement factors in BAL (proteomic analysis) and plasma (TCC) between IPF patients and controls was performed with MANCOVA using complement factors as dependent, diagnosis (IPF or control) as fixed and age, sex and BMI as covariates. Spearman correlation was used to assess associations between proteomic components and BMI as well as with TCC within patients dichotomized at TCC 0.7 CAU/mL.

### Ethical considerations

The study was approved by the Regional Committee for Medical Research Ethics (2013/2358) and written informed consent was obtained from all participants.

## Results

**Figure 1:**
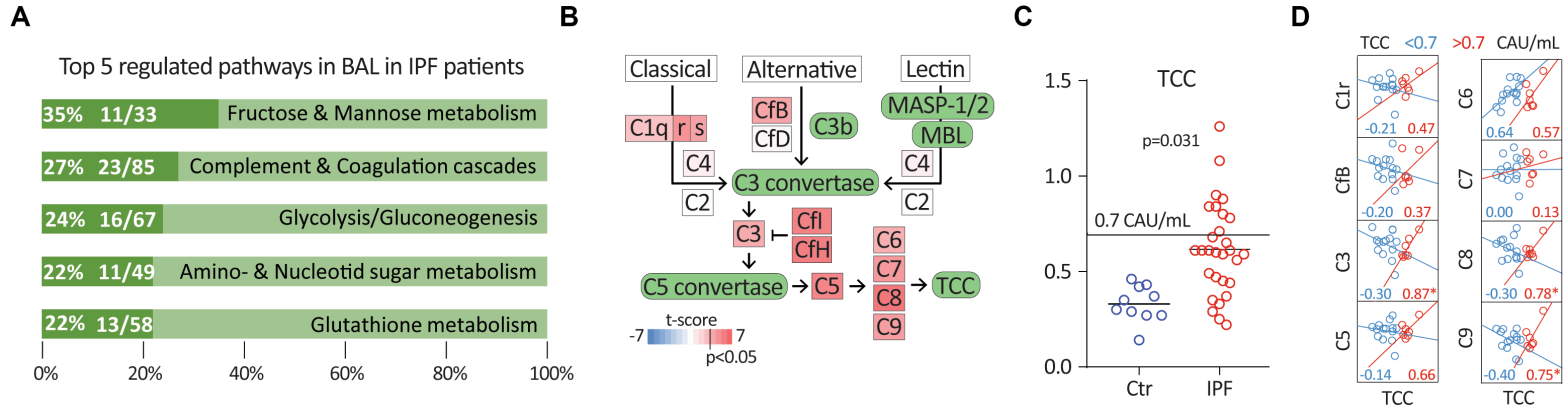
**(A)** Top 5 regulated pathways in BAL in IPF patients using KEGG pathway analysis. **(B)** Scheme of the complement system. Differentially regulated complement components (*p* < 0.05) marked in shades of red colour, i.e., upregulated; depending on the t-score after the proteomics analysis (IPF patients vs. controls). **(C)** Terminal complement complex (TCC) in EDTA plasma in controls (Ctr) vs. IPF [complement activation unit (CAU)/mL]. **(D)** Correlation plots and r-value between complement factors, C1r, CfB, C3, C5, C6, C7, C8 and C9 and TCC < 0.7 and TCC > 0.7 in IPF patients only. *p < 0.05.

In the proteomic dataset, we found 567 differentially regulated proteins (118 upregulated and 449 downregulated) (*p* < 0.05, 0.66 > FoldChange >1.5) in BAL, comparing IPF patients with controls. Pathway analyses of the 567 proteins are listed in Figure 1, showing the top 5 significantly enriched pathways (with >10 proteins present) in IPF compared to controls (Figure 1A). The complement and coagulation system were among the pathways with most proteins differentially regulated in IPF as compared with controls, with 23 out of 85 proteins involved (27%) (Figure 1A). The proteins C5, C6, C7, C8, and C9, all part of the complement end product, TCC, were all significantly upregulated (Figure 1B). In addition, we found that several of the complement proteins in the classical pathway and alternative pathways, such as C1r and complement factor B, were upregulated.

Focusing on TCC, C1r, complement factor B and C3, C5-9, these factors correlated well with BMI (all r between 0.43 and 0.64, *p* < 0.05), except TCC (*r* = −0.28, *p* = 0.14) and C6 (*r* = 0.14, *p* = 0.52). Table 2 lists the complement components from the proteomics analysis (IPF patients vs. controls) with age, sex and BMI adjusted *p*-values.

**Table 2:** Complement component proteomic data in BAL comparing patients with IPF vs. Controls.

UniProt	Gene name	Protein name	Fold change IPF vs. Ctr	Adj P^*^
B4DPQ0	C1R	Complement C1r subcomponent	2.46	<0.001
Q9NZP8	C1RL	Complement C1r subcomponent-like protein	1.52	0.010
P02745	C1QA	Complement C1q subcomponent subunit A	0.8	0.074
A0A0A0MSV6	C1QB	Complement C1q subcomponent subunit B	0.4	0.010
P02747	C1QC	Complement C1q subcomponent subunit C	0.7	0.030
P09871	C1S	Complement C1s subcomponent	0.4	<0.001
Q9NPY3	CD93	Complement component C1q receptor	2.51	<0.001
P06681	C2	Complement C2	1.1	0.94
P01024	C3	Complement C3	1.71	<0.001
P0C0L5	C4B	Complement C4-B	0.7	0.56
P01031	C5	Complement C5	2.83	<0.001
P13671	C6	Complement component C6	1.87	0.001
A0A0D9SEN1	C7	Complement component C7	3.20	<0.001
P07357	C8A	Complement component C8 alpha chain	3.47	<0.001
P07358	C8B	Complement component C8 beta chain	2.44	<0.001
P07360	C8G	Complement component C8 gamma chain	2.97	<0.001
P02748	C9	Complement component C9; C9a; C9b	3.20	<0.001
B4E1Z4	CFB	Complement factor B	1.81	<0.001
K7ERG9	CFD	Complement factor D	1.0	0.77
P08603	CFH	Complement factor H	3.75	<0.001
Q03591	CFHR1	Complement factor H-related protein 1	5.22	0.005
G3XAM2	CFI	Complement factor I; heavy chain;light chain	2.28	<0.001

^*^Adjusted for age, sex and BMI.

After adjustment for age, sex and BMI, IPF patients had significantly increased TCC levels in plasma compared to controls [mean (SD) 0.62 (0.24) vs. 0.33 (0.10), *p* = 0.031] (Figure 1C). Elevated TCC plasma values (≥0.7 CAU/mL) were present in 34% of the IPF patients, but in none of the controls. Of the IPF patients, 72% had TCC levels higher than the controls. In patients with elevated plasma TCC (>0.7 CAU/mL), TCC was significantly correlated with the complement factors, C3, C8, and C9 in BAL (Figure 1D). We found no correlation between lung function (FVC and DLCO) and TCC levels in plasma.

## Discussion

Our results suggest that complement plays a role in the pathogenesis of IPF. Proteomic analysis of BAL demonstrated that the complement system was highly differentially regulated in IPF patients as compared with controls. Importantly, all five components of TCC were upregulated in the proteomic analyses. TCC in plasma was also elevated in IPF patients as compared to controls, and elevated levels of TCC correlated with C3, C8, and C9 complement factors in BAL.

Proteomic analysis of BAL revealed that the complement and coagulation cascade were the top two differentially regulated pathways, with half of the proteins in the complement pathway elevated in IPF patients as compared with controls. Similarly, in a proteomic analysis of peripheral blood, comparing IPF patients to normal controls, O’Dwyer et al. found that complement and coagulation cascade were among the most significantly enriched clusters (15). Gu et al. have identified C5b-9 in fibroblasts in lung explants of IPF patients who have undergone lung transplantation, as well as elevated levels of TCC in BAL and plasma compared to controls (16). They found that blocking complement receptors C3aR and C5aR stopped the progression of animal experimental bleomycin-induced lung fibrosis and suppressed the local complement activation, which indicate that complement plays a role in disease progression (16). Meliconi et al. found elevated levels of C3d, C4d, and Factor Ba in humans, demonstrating that conversion products of all complement pathways are increased in IPF (17). Higher C3 expression is also associated with a MUC5B promoter variant that has been shown to be a strong risk factor for the development of IPF, suggesting that it may contribute to the pathogenesis of IPF (18). Also, IL17-A and transforming growth factor beta (TGFβ) have been suggested to have a role in complement activation and in the pathogenesis of IPF (19, 20). IL17A mediates epithelial injury via TGFβ that again downregulates Complement Inhibitory Proteins (CIP), leading to complement activation.

The proteomics analyses do not measure activated products of the complement system, but rather native complement components present in the lung. However, elevated levels of plasma TCC in IPF clearly indicate that systemic complement activation occurs in these patients. The proteomics data indicate that there are sufficient amounts of all components needed to activate the terminal pathway to form TCC, and it is reasonable to assume a local activation in the IPF lung. When TCC is formed, the small potent peptide C5a is released. C5a is one of the most important contributors to inflammation, inducing innumerable cytokines and other inflammatory mediators (21). In accordance with the findings of Gu et al. (16), our findings support a role of the C5a-C5aR axis as a possible driver of chronic inflammation and progression of fibrosis. The increased plasma concentration of TCC may be due to leakage of TCC from the lungs to the circulation, or reflect a low-grade systemic activation occurring in these patients, or a combination thereof. We were unable to demonstrate any correlation between complement components (in the lung) and lung function parameters.

The study has limitations. We were not able to match the control group with the IPF-group with respect to age and sex. Since the two groups were not properly matched, we controlled for age and sex in the statistical analyses. In addition, many of the complement factors correlated with BMI, and BMI was therefore also included as an adjustment variable. In a study that involves bronchoscopy and BAL, it is difficult to recruit healthy controls. For practical and ethical reasons, we chose to recruit controls among subjects who should undergo bronchoscopy anyway, as part of annual surveillance after curative resection of carcinoid tumors. They were non-smokers and considered healthy with no previous or current medication that could interfere with the results.

In conclusion, IPF was associated with upregulation of terminal complement components in BAL, and increased terminal complement complex in plasma, suggesting a role for the complement system in the pathogenesis of IPF.

## Data availability statement

The mass spectrometry proteomics data to the ProteomeXchange Consortium is available via the PRIDE partner repository with the dataset identifier PXD036638. In the deposited file, there are also mass spectrometry data from patients with sarcoidosis and hypersensitivity pneumonia, which are not included in the current study with only IPF patients and controls. In the supplement of the present paper, the mass spectrometry data is available as a raw data file (excel file) with analysis from Perseus.

## Ethics statement

The studies involving human participants were reviewed and approved by the Regional Committee for Medical Research Ethics (2013/2358). The patients/participants provided their written informed consent to participate in this study.

## Author contributions

LS and TU participated in data collection and performed the laboratory work. All authors contributed to the design of the study, interpretation of the results, and drafting and revising the manuscript and approved the final version.

## Funding

This study received funding from Norwegian Respiratory Society sponsored by Boehringer Ingelheim. The funder was not involved in the study design, collection, analysis, interpretation of data, the writing of this article or the decision to submit it for publication.

## Conflict of interest

MTD has received research funding (to his institution) and speaker/consulting fees from Boehringer Ingelheim and Roche, unrelated to the current study.

The remaining authors declare that the research was conducted in the absence of any commercial or financial relationships that could be construed as a potential conflict of interest.

## Publisher’s note

All claims expressed in this article are solely those of the authors and do not necessarily represent those of their affiliated organizations, or those of the publisher, the editors and the reviewers. Any product that may be evaluated in this article, or claim that may be made by its manufacturer, is not guaranteed or endorsed by the publisher.
