# Supplementary material for: A role for the terminal C5-C9 complement pathway in idiopathic pulmonary fibrosis
Source: Front Med (Lausanne). 2023 Aug 9;10:1236495. doi: 10.3389/fmed.2023.1236495 (PMC10444977; doi:10.3389/fmed.2023.1236495)
Supplement: Supplementary file 1 [file Data_Sheet_1.PDF]

Supplementary material: Analysis of TCC

Materials:

Microtiterplates for EIA: Nunc Maxisorp F96 or Costar.

Antibodies:

1. Coat: Mouse anti-human SC5b-9 (aE11) ascites, clone 95B, (stock: 3mg/mL).
2. Secondary antibody: Mouse anti-human C6 biotinylated (clone 9C4, Batch 9C4-B, 1mg/ml, 24.08.2007).
3. Conjugate: Streptavidin-horseradish peroxidase (Amersham RPN 1231).

Substrate:

ABTS/H_2O_2/acetate buffer (0.15M, pH 4.0): 180mg ABTS/ l acetate buffer.

Add 3% H_2O_2 1:1000 to the ABTS-solution shortly before adding substrate.

Standard:

INTERNATIONAL COMPLEMENT STD - ICS Batch 2. (ICS#2)

Zymosan and Heath Aggregated IgG (HAIgG) activated normal human serum, defined as 1000 CAU/mL. (Complement Arbitrary Units).

Samples:

EDTA-plasma

Buffers:

1.coating buffer:	PBS pH 7.4
2.washing buffer:	PBS, 0.1 % tween 20
3.sample buffer:	PBS, 0.2 % tween 20, 10 mM EDTA
4.antibody buffer	PBS, 0.2 % tween 20
5.substrate buffer:	0.15 M Na-acetate buffer, pH 4.0

METHOD:

1. Coat the plate with 100 µl of aE11 ascites diluted 1:10 000 in buffer 1. Incubate for up to 7 days at 4°C (or overnight at room temperature) with plastic sealing or moisture chamber
2. Wash x 3 in buffer 2.
3. Add 100 µl standard or sample with appropriate dilutions. Incubate for 1h at 4°C.
   Standard: ICS #2: Dilute 1st step 1:200 in buffer 3 (5 µl ICS#2 in 1000 µl, giving a conc. of 5 CAU/mL). Make a serial dilution with 500 µl in 6 following tubes, ending up with 7 tubes (tube no 2 can be omitted to save space on the plate, but step 1 and 3-7 must be included – giving a standard with 6 steps).
   Samples: Dilute normal plasma 1:5 in buffer 3. Highly activated samples may need further dilution.
4. Wash x 3 in buffer 2.
5. Add 100 µl mouse anti- human C6 biotinylated (clone 9C4), diluted 1:2500 in buffer 4. Incubate for 45 min. at 37°C.
6. Wash x 3 in buffer 2.
7. Add 100 µl Streptavidin-horseradish peroxidase (dilute 1:1000 or as recommended from the Supplier, in buffer 4). Incubate for 45 min. at 37°C.
8. Wash x 3 in buffer 2.
9. Add 3% H_2O_2 1:1000 to the ABTS-solution (e.g. 12 µl to 12 ml for one plate) shortly before adding substrate to plate (keep in dark if not immediately added).
10. Add 100 µl substrate, read at 405 nm ref. 490 nm after 10 – 20 min. (or when OD at std 1 >0.7).
